# Clinical and cost-effectiveness of computerised cognitive behavioural therapy for depression in primary care: Design of a randomised trial

**DOI:** 10.1186/1471-2458-8-224

**Published:** 2008-06-30

**Authors:** L Esther de Graaf, Sylvia AH Gerhards, Silvia MAA Evers, Arnoud Arntz, Heleen Riper, Johan L Severens, Guy Widdershoven, Job FM Metsemakers, Marcus JH Huibers

**Affiliations:** 1 Department of Clinical Psychological Science, Faculty of Psychology, Maastricht University, The Netherlands; 2 Department of Health Organization, Policy and Economics, Faculty of Health, Medicine and Life Sciences, Maastricht University, The Netherlands; 3 Trimbos-institute, Utrecht, The Netherlands; 4 Department of Clinical Epidemiology and Medical Technology Assessment, University Hospital Maastricht, The Netherlands; 5 Department of Health, Ethics and Society/Metamedica, Faculty of Health, Medicine and Life Sciences, Maastricht University, The Netherlands; 6 Department of General Practice, Faculty of Health, Medicine and Life Sciences, Maastricht University, The Netherlands

## Abstract

**Background:**

Major depression is a common mental health problem in the general population, associated with a substantial impact on quality of life and societal costs. However, many depressed patients in primary care do not receive the care they need. Reason for this is that pharmacotherapy is only effective in severely depressed patients and psychological treatments in primary care are scarce and costly. A more feasible treatment in primary care might be computerised cognitive behavioural therapy. This can be a self-help computer program based on the principles of cognitive behavioural therapy. Although previous studies suggest that computerised cognitive behavioural therapy is effective, more research is necessary. Therefore, the objective of the current study is to evaluate the (cost-) effectiveness of online computerised cognitive behavioural therapy for depression in primary care.

**Methods/Design:**

In a randomised trial we will compare (a) computerised cognitive behavioural therapy with (b) treatment as usual by a GP, and (c) computerised cognitive behavioural therapy in combination with usual GP care. Three hundred mild to moderately depressed patients (aged 18–65) will be recruited in the general population by means of a large-scale Internet-based screening (*N *= 200,000). Patients will be randomly allocated to one of the three treatment groups. Primary outcome measure of the clinical evaluation is the severity of depression. Other outcomes include psychological distress, social functioning, and dysfunctional beliefs. The economic evaluation will be performed from a societal perspective, in which all costs will be related to clinical effectiveness and health-related quality of life. All outcome assessments will take place on the Internet at baseline, two, three, six, nine, and twelve months. Costs are measured on a monthly basis. A time horizon of one year will be used without long-term extrapolation of either costs or quality of life.

**Discussion:**

Although computerised cognitive behavioural therapy is a promising treatment for depression in primary care, more research is needed. The effectiveness of online computerised cognitive behavioural therapy without support remains to be evaluated as well as the effects of computerised cognitive behavioural therapy in combination with usual GP care. Economic evaluation is also needed. Methodological strengths and weaknesses are discussed.

**Trial registration:**

The study has been registered at the Netherlands Trial Register, part of the Dutch Cochrane Centre (ISRCTN47481236).

## Background

Major depression is a common mental health problem in the general population [1] and it is reported to be the second most common and costly mental health problem in general practice [2]. Depression is associated with substantial decreases in quality of life through its impact on physical, social and emotional functioning, and well-being [3,4]. By 2020, depression is estimated to be the second leading contributor to the global burden of disease [5]. Cost-of-illness studies reveal that the economic burden of depression is considerable [6-8].

### Difficulties in the treatment of depression in primary care

The general practitioner (GP) is the major health care provider involved in the primary care treatment of depression. In the Dutch health care system the GP is seen as a gatekeeper, and as a result patients view their GP as a key figure in the detection and treatment of depression [9]. Despite this, many depressed patients remain undetected [10-12]. Even when the depressive complaints are being recognised, many patients in primary care do not receive the care they need. There are several reasons for this. First, time for the management of psychosocial problems is lacking [13]. Second, pharmacotherapy is only effective in extremely depressed patients [14], and many patients refuse medication or comply poorly [15]. Third, effective non-pharmacological treatment options, such as psychotherapy, in primary care are scarce or not feasible [16]. Consequently, only a limited group of depressed patients in primary care receives effective treatment.

### Computerised cognitive behavioural therapy in primary care

Cognitive behavioural therapy (CBT) is one the most widely researched forms of psychotherapy. Cognitive behavioural therapy (CBT) has proven to be as effective as pharmacotherapy in the acute phase of mild to severe depression, and seems even more effective in the prevention of recurrence and relapse [17-19]. Despite its effectiveness, face-to-face CBT in primary care has some major limitations. There are not enough well trained therapists, it is costly, there are waiting lists, and patients may feel reluctant to enter psychotherapy. An alternative treatment in primary care might be computerised cognitive behavioural therapy (CCBT): a computer program based on the principles of CBT. The level of therapist support can vary considerably in CCBT. It can be offered as a self-help intervention without or with only minimal support. Previously, written self-help based on CBT seemed a promising treatment for depression [20]. In a primary care setting, positive outcomes were found regarding the (cost-) effectiveness of written self-help with minimal contact in subthreshold depression relative to care as usual provided by the GP [21,22].

CCBT for primary care seems promising; it provides an acceptable alternative to pharmacotherapy, it can save clinicians' time, and the costs are low compared with face-to-face CBT. Furthermore, CCBT has a high accessibility, the number of referrals to secondary care by a GP can be reduced, and waiting lists for traditional CBT can become shorter [23,24]. Next to that, CCBT may fit very well in a stepped care program, and may function as a first step in the treatment of depression [25]. In a recent systematic review [23], it was concluded that CCBT is a feasible, effective and acceptable treatment for depression. However, most research on the efficacy of CCBT has been conducted in the general population or within clinical or specialist settings. To our knowledge, only one study, so far, investigated the efficacy of CCBT for depression in primary care [26], and it was shown that CCBT (delivered on a personal computer in the general practice) is more effective than usual care by a GP in mild to moderate depression and anxiety. Furthermore, this intervention seemed promising regarding the cost-effectiveness compared with usual GP care. CCBT was both more effective and more costly compared with usual GP care. When willing to pay for an additional unit of effect, CCBT could be very cost-effective. If a value of £40 is placed on a unit reduction on the Beck Depression Inventory, the probability of CCBT being cost-effective is in excess of 80%. At a value of £5000 for 1 quality-adjusted life-year (QALY), the study showed that there is an 85% chance of CCBT being more cost-effective, and at a value of £15000 per QALY it exceeds a 99% chance of being cost-effective [27]. Nevertheless, more research is necessary; so far only this one study has conducted an economic evaluation of CCBT, and the effects of CCBT in combination with usual care by a GP are still unknown. In addition the efficacy of CCBT via the Internet in primary care remains to be evaluated. The Internet can offer further advantages in comparison to CCBT on a stand-alone computer; it is easily accessible and it can be used at home, anonymously, and it is available 24/7.

### Current study

In the present study we aim to evaluate the (cost-) effectiveness of online CCBT for mild to moderate depression in primary care. In a randomised trial we will compare (a) CCBT with (b) treatment as usual (TAU) by a GP, and (c) CCBT in combination with TAU. In a recent Dutch study of Spek et al. (2007), the same CCBT program has shown to be equally effective as group CBT in people over 50 years old with subthreshold depression [28]. Based on the results from a recent systematic review [23] we hypothesise that CCBT will be more effective than TAU by a GP. Furthermore, we hypothesise that CCBT in combination with TAU will be more effective than CCBT alone by increasing treatment adherence. Although some studies have shown that for mild depression a combination of pharmacotherapy and psychotherapy does not appear more effective than psychotherapy alone [29], other studies suggested that patients receiving combined treatments were more likely to stay in treatment and comply to the treatment protocol [30].

Regarding the economic evaluation we hypothesise the following. The self-help intervention CCBT alone implies costs of time spent by the patient on the treatment and costs of the development of the program, while the TAU treatment requires costs related to a GP consult and/or medication. We hypothesise that from a societal perspective, these costs of the intervention CCBT are comparable to the costs of the intervention TAU. However, as a consequence of our hypothesis that CCBT is more effective than TAU, we expect that CCBT will be more preventive in health care use and productivity loss during the follow-up period, and thus result in lower costs compared with TAU by a GP. As a consequence of the expected increase of effectiveness (in terms of depression and quality of life) and decrease of costs, we hypothesise that CCBT is more cost-effective than TAU by a GP. Hypotheses about the intervention CCBT in combination with TAU are that it is both more costly and more effective compared with stand alone CCBT or TAU.

## Methods

### Design

**Figure 1:**
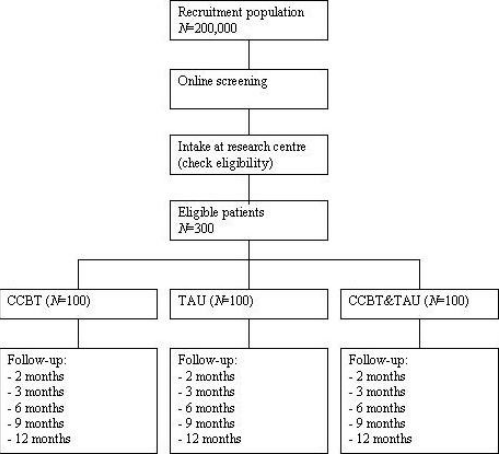
Flow of the participants.

We will conduct a randomised controlled trial. Patients will be randomly allocated to one of the three following conditions: (a) CCBT, (b) TAU by a GP, and (c) a combination of CCBT and TAU by a GP. The design of the study and the anticipated flow of the participants are graphically shown in Figure 1. The Medical and Ethical Committee of Maastricht University approved the study protocol. The study is registered at the Netherlands Trial Register, part of the Dutch Cochrane Centre (ISRCTN47481236).

### Study population

The patient population we aim to investigate consists of 300 mild to moderately depressed adults. Patients are eligible to participate if they meet the following criteria: age 18 to 65 years; access to the Internet at home; at least mild to moderate depressive complaints (BDI-II score > 16), although a DSM-III-R diagnosis of major depression was not required; duration of depressive complaints 3 months or more; no current psychological treatment for depression; no continuous antidepressant treatment for 3 months or more prior to entry; fluent in Dutch language; no alcohol and/or drug dependence; and no severe psychiatric co-morbidity (e.g. psychotic disorders).

### Sample size

Power calculations are based on elementary head-to-head comparisons of CCBT versus usual care and CCBT versus the combined treatment (t-test). A clinically relevant treatment effect is derived from the only study of CCBT in primary care [26]. For a mean difference in change scores of 5 (SD 5.25) on the BDI-II, a sample size of 84 participants per group is needed (power 90%, alpha 0.05). Adjusting for potential study withdrawal (20%), we estimate that 100 participants per group are needed.

### Recruitment

Participants will be recruited in the general population by means of a large-scale Internet-based screening in the South of the Netherlands. A random selection of individuals (*N *= 200,000) will be sent an invitation letter to complete a screening questionnaire (i.e. BDI for primary care) via the Internet. The letters will be sent in weekly badges. Six municipalities cooperate by issuing names and addresses of their residents on a weekly base. The letters will contain information on the study protocol as well as log in codes for the questionnaires on the Internet. Participants who score above the cut-off score of four on the BDI for primary care [31] will be invited to visit the research centre for an interview where final eligibility will be assessed based on the in- and exclusion criteria. After inclusion we will notify the patients' GP about his or her participation in the study.

### Randomisation and procedure

Randomisation will take place after informed consent is obtained. An independent statistician will develop the randomization code. Block randomization will be used to ascertain that each intervention consists of equally large groups. The randomization code will be given to an independent IT-specialist who will develop a computer program to carry out the group allocation. On entry into the trial the computer program provides the next available number. The randomization code will not be revealed until participant inclusion is complete. In view of the nature of the treatments, blinding of the participants and researchers is not possible.

Baseline assessment will take place at the research centre before randomization. The questionnaires will be administered on a computer. All follow-up assessments will take place at home via the Internet at two, three, six, nine and twelve months after inclusion. Preceding an upcoming assessment point, participants will receive an email alert. If a participant fails to complete the assessment within one week, an email-reminder will be sent. When the participant still fails to fill out the questionnaires, a phone call will be made.

Individuals will not be paid for participation, but will receive a small fee for Internet use.

### Interventions

The CCBT program (named "colour-your-life") is an online, multimedia, interactive, self-help computer program for depression, and was developed by Riper and Kramer (2004) [32] of the Trimbos-institute (the Netherlands Institute of Mental Health and Addiction). The program is based on the principles of CBT and on the Dutch version of the 'coping with depression course' of Lewinsohn [33,34]. The program consists of eight weekly 30-minute sessions and a ninth booster session, although the duration of sessions can vary among users. At the end of each session homework-assignments are given, such as keeping a 'mood diary'. Patients allocated to the intervention CCBT or the combination CCBT and TAU will be given log in codes by the researchers at inclusion and they will access the program at home. No professional assistance will be offered. The program was originally developed for people over 50-years old [28] and is adapted for an adult population (18 to 65 years) for the current study. GP's of patients allocated to CCBT only will be informed about the participation and treatment allocation of their patient.

In the TAU and CCBT&TAU group, participants will be advised to contact their own GP. GPs of patients in the TAU and the CCBT&TAU group will be sent a letter, informing them about their patients' study participation, and advising them to follow the depression guideline as described by the Dutch College of General Practitioners [35]. This guideline states that treatment should formally consist of four to five biweekly consultations over the course of nine weeks in combination with antidepressant treatment if indicated. In case of suicidal risk, social dysfunction, symptom deterioration or non-improvement in six to twelve weeks time, referral to specialist mental health care settings is recommended. In practice however, usual care is whatever the GP prescribes.

An integrity check will be performed to assess treatment quality and protocol compliance using computer records of the CCBT program and questionnaires assessing use of GP and other health care (i.e. health care use questionnaire).

### Instruments

Instruments will be used for the screening process, the clinical outcome assessment and the economic evaluation. In Table 1 an overview of all assessments per time point is shown.

**Table 1:** Overview of instruments per time point

**Instrument**	**Screening**	**Baseline**	**2 months**	**3 months**	**6 months**	**9 months**	**12 months**
Demographic variables	X						
Beck Depression Inventory PC	X						
Composite International Diagnostic Interview		X					
Beck Depression Inventory II		X	X	X	X	X	X
Symptom Checklist 90		X		X			X
Work and Social Adjustment Scale		X		X			X
Dysfunctional Attitudes Scale		X	X	X	X	X	X
WHOQoL-BREF		X		X			X
EuroQol		X		X	X	X	X
Productivity and disease questionnaire		X		X	X	X	X

*Note*. In addition to the regular follow-up measurements, each month the health care use questionnaire will be administered.

#### Beck Depression Inventory for Primary Care

The Beck Depression Inventory for Primary Care (BDI-PC) is a screening instrument for depression consisting of seven items derived from the Beck Depression Inventory II. Each item is assessed at a 4-point Likert-scale (range 0 – 4). Several studies have proven its sound psychometric properties [31,36,37]. A cut-off score of 4 was used in the current study, since this has excellent sensitivity and specificity for identifying a diagnosis of major depression [31].

#### Composite International Diagnostic Interview

To determine in- and exclusion criteria the computerised Composite International Diagnostic Interview (CIDI-auto) will be used. The CIDI is an extensive, fully structured diagnostic interview to assess lifetime and 12-month DSM-III-R diagnoses. The CIDI can be used by lay-interviewers and has acceptable reliability and validity [38,39].

#### Beck Depression Inventory Second Edition

The Beck Depression Inventory II (BDI-II) will be used to measure depressive severity. The total score is the sum of the 21 items with a range of 0 (no depression) to 63 (severe depression). There has been consistent support for the construct validity and reliability of the BDI-II in various samples [40-42].

#### Functional impairment

Functional impairment will be assessed with the SF-36 Health Survey (SF-36), which consists of 36 items measuring 8 multi-item scales. For each subscale items are coded, summed, and transformed on to a scale from 0 (worst possible health state measured by the questionnaire) to 100 (best possible health state). The SF-36 has good psychometric properties in terms of validity, reliability, and scale structure [43-45]. Additionally, the economic evaluation will use the SF-6D utility, which is a quality of life measure derived from the SF-36 [46].

#### Psychological distress

The Symptom Checklist (SCL-90) is a self-report symptom inventory of psychopathology. The SCL-90 consists of 90 items, each rated on a five-point scale of distress from 'not at all' to 'extremely' [47,48]. The total score of the SCL-90 can be used as an index of severity for general psychological distress (score range 90–450) [49]. The SCL-90 has a high degree of reliability and support has been found for the validity [47].

#### Social functioning

The Work and Social Adjustment Scale (WSAS) is a self-report scale of functional impairment attributable to an identified problem. The WSAS consists of five items measured on an 8-point Likert-scale (0 to 8). A high score indicates severe impairment. It has good psychometric properties [50].

#### Dysfunctional attitudes

The Dysfunctional Attitude Scale form A (DAS-A) is a self-report scale designed to measure the presence and intensity of dysfunctional attitudes. The DAS-A consists of 40 items and each item consists of a statement and a 7-point Likert-scale (7 = fully agree; 1 = fully disagree). The higher the score, the more dysfunctional attitudes an individual reports [51].

#### Quality of life measures for the economic evaluation

Measuring health-related quality of life is relevant in patients with depression, since depression has a large impact on the physical, social and emotional aspects that are relevant and important to a patient's well-being [3,4]. An intervention aimed at treating depression is therefore expected to have an impact on the perceived quality of life. In addition, measuring generic quality of life facilitates the comparison of the effects on quality of life of our intervention program with that of other interventions [52]. Quality of life will be measured in three different ways in this study by using the WHOQOL-BREF, the EuroQol and the SF-6D.

The WHOQOL includes a broader range of mental health aspects in its psychological domain compared to other quality of life instruments and is therefore more feasible to use in this study [53]. The WHOQOL-BREF is an abbreviated version of the WHOQOL-100, which has proven to be a valid and reliable alternative [54,55]. The WHOQOL-BREF measures four domains related to quality of life (physical health, psychological health, social relationships and environment) and includes two questions on overall quality of life and general health [55].

Generic quality of life will be derived by means of the EQ-5D of the EuroQol group. The EQ-5D consists of five health state dimensions (mobility, self-care, usual activity, pain/discomfort and anxiety/depression) on which the respondent has to indicate his own health state [56,57]. The EuroQol will be assessed since it is a validated and widely used quality of life instrument, both nationally and internationally [58]. An advantage of the EuroQol is that it is short and that an overall utility score for population-based quality of life can be obtained, which facilitates comparisons with other interventions and health states in other disease areas. A utility refers to the preference that individuals or society may have for any particular set of health outcomes. It is indicated by a number between 0 (the worst imaginable condition: death) and 1 (perfect health) [52]. Standardised value sets are available to calculate the utility based on the EQ-5D. This study will use the Dutch tariff and the original UK tariff to value generic quality of life [58-60]. The utility scores of the EQ-5D will be used to calculate the quality adjusted life year (QALY) during the follow-up period by adjusting the length of time affected through the health outcome by the utility value [52].

An adapted version of the EuroQol is the EQ-5D+C, in which a sixth domain (cognitive functions referring to memory, concentration, coherence and IQ-level) is added to the five existing domains of the EQ-5D [61-63]. This sixth domain of the EQ-5D+C is also included in the questionnaire to provide additional information on quality of life. However, since there is no tariff developed to compute utility scores for the EQ-5D+C, utility scores and QALYs will only be calculated based on the five domains of the EQ-5D.

The SF-6D is a utility instrument based on the health-related quality of life questionnaire SF-36. The utility score is derived from 11 items of the SF-36 and is composed of six dimensions of health (physical functioning, role limitations, social functioning, pain, mental health, and vitality). The SF-6D utilities will be derived by means of the preference-based UK tariff [46].

#### Costs

Costs will be defined from the societal perspective. Within our study we distinguish three cost-categories: health care sector costs, costs for the patient and family, and productivity costs [52].

A health care use questionnaire will be developed to measure the psychological, paramedical, medical, paid and informal care, participation in a self-help group, and alternative treatments received by the patient on a monthly base. This health care use questionnaire will be based on an existing cost diary [64] and retrospective cost questionnaires [65,66], and is adapted to depressive patients. This questionnaire will provide information to calculate health care sector costs by measuring the volume of care provided, and out-of-pocket expenses, which are part of the costs for the patient and family. The other part of the patient and family costs will concern costs of travelling and lost time due to the primary care intervention TAU and/or CCBT. The time spent by a patient on CCBT will be tracked by means of the computer-registered login and logout data of the program. In the health care use questionnaire the duration of a GP consult will be registered by the patient. The number of GP consults informs on the number of travels from/to the GP, and will be linked to average distances from/to a GP. Dutch guideline prices will be used to value the costs of the health care items [67]. If for specific cost-categories cost guidelines are unavailable, average tariffs or shadow prices will be used. The standard cost prices and tariffs of health care practitioners include the integral costs, being all costs directly and indirectly attributable to the cost unit.

For the measurement of production losses, the patient modules B, C, D and E of the PROductivity and DISease Questionnaire (PRODISQ) will be used [68,69]. These modules consist of questions concerning the profession, working situation, income, absence from work, compensation mechanisms in case of absence for paid work and productivity costs at work (efficiency loss) of the patient. Productivity costs will be calculated according to the friction cost approach [67,70], using one general cost price per lost hour of productivity [67]

### Analyses

#### Clinical analyses

Data-analysis will include intention-to-treat analysis and per-protocol analysis. Analysis will include elementary head-to-head comparisons of the intervention groups as well as more complicated multi-level analysis incorporating patients and time measurements if necessary. An integrity check and a process evaluation will be performed using qualitative methods of analysis. In case of missing data, we will impute intermittent missing data using the mean of the values of a previous and a subsequent time point. Other missing data (i.e. missing values due to lost to follow-up) will only be imputed when more then 15% of the data are missing.

To test the main hypotheses, difference scores for all outcome variables will be calculated (t_0 _minus t_k_) and compared between the three groups using ANOVA. In case of violation of assumptions, robust ANOVA can used [71]. We will then compute improvement effect sizes and between-group effect sizes [72] or robust equivalents [see [73]]. Next, we will calculate the number of patients who showed reliable and clinically significant change on the BDI-II using the method of Jacobson and Truax [74]. This calculation is based on two components: (1) the extent to which the pre-to-post-difference score is reliable taking into account the measurement variability of the instrument (reliable change; RC), and (2) the extent to which post-treatments scores are clinically meaningful (clinically significant change; CSC) [75]. Chi-square tests will be used to test the frequency differences in RC, CSC and RC+CSC between the three groups.

#### Economic evaluation

Since the follow-up period lasts one year and no extrapolation over time will be executed, discounting of costs is not necessary. All costs will be indexed to the year 2007 by means of the price indexes of the Dutch Central Bureau of Statistics (CBS).

For each patient, volumes of care, travels, lost time for receiving care and lost productivity hours will be multiplied by the prices determined for each cost item. Based on the costs per item, costs during the follow-up period will be calculated as the cumulative costs per patient. The costs at baseline and during the follow-up period of the three groups will be compared by the non-parametric bootstrapping method with confidence intervals in percentiles. By bootstrapping, samples of the same size as the original data are drawn with replacement from the observed data [76]. The quality of life and clinical outcome variables will be compared between the three groups at baseline and during the follow-up period using ANOVA.

In case of baseline differences of costs, effectiveness, or utility scores between the patient groups, corrections will be performed [77-79]. The economic evaluation will consist of a base-case cost-effectiveness and cost-utility analysis, and sensitivity analyses. Uncertainty of parameter estimates of the base-cases will be dealt with by these sensitivity analyses [52]. In the base-cases, data will be analysed according to the intention-to-treat principle. Incremental cost-effectiveness ratios (ICERs) will be determined on the basis of incremental costs and incremental effects [52] of (a) stand alone CCBT compared with (b) usual GP care, or (c) a combination of CCBT and usual GP care. The primary outcome measure for the cost-effectiveness analysis is depression measured by the BDI-II, and for the cost-utility analysis the QALY based on the EQ-5D. The cost-effectiveness ratio will be stated in terms of costs per point improvement on the BDI-II, the cost-utility ratio will focus on the net cost per QALY gained. In our primary analysis QALYs will be derived from the EQ-5D using the UK-tariff. Scores on the quality of life domains derived from the WHOQOL-BREF and the sixth domain of the EQ-5D+C will be used to provide in-depth insight into the quality-of-life.

Non-parametric bootstrap re-sampling techniques will be used to explore uncertainty around estimates of cost-effectiveness derived from the study sample [76]. Decision uncertainty will be represented graphically by means of a cost-effectiveness acceptability curve (CEAC) [52,80,81]. In addition, the net monetary benefit (NMB) will be used to present the cost-effectiveness and cost-utility results in monetary units. The NMB expresses the difference in effects between the intervention groups in monetary values using the threshold willingness-to-pay for a unit of effect, minus the difference in costs between the interventions [52,82]. Since the value that society would place on a unit reduction in BDI-II depression score is unknown, different values will be assumed to calculate the NMB [27]. Regarding the QALY, the Dutch Council of Public Health and Care suggested in 2006 a ceiling of £80.000 per QALY per year [83]. Alternative values, ranging to £80.000 per QALY, will be used to estimate the NMB.

The alternative threshold values of the NMB will be analysed in sensitivity analyses. Other aspects that can be part of a sensitivity analysis are: varying the utility outcome measure by using the SF-6D or alternative tariffs to value the EQ-5D utility (Dutch tariff instead of the UK tariff), or including other effectiveness measures used in the clinical evaluation. Cost prices will be varied as minimum and maximum cost price estimates. Next to the intention-to-treat analysis, a per-protocol analysis can be performed.

### Collaboration

The current study will be conducted in collaboration with several disciplines. The Trimbos-institute (the Netherlands Institute of Mental Health and Addiction) will be involved in the development and dissemination of CCBT. The Pandora foundation (patient organization) acts as an advisor on the design of the study and the dissemination of the results. Several members of the project group work as clinicians in secondary mental health care institutions and have substantial professional contacts in the field. The

Dutch College of General Practitioners (NHG) has been informed about our plans and supports our initiative. Additional research projects will be conducted in collaboration with the Department of Health, Ethics and Society/Metamedica and the Department of Health Organization, Policy and Economics of the Maastricht University. For instance, these projects will focus on topics like the patient perspective on CCBT and quality of life aspects of depression.

## Discussion

Although in the last two decades research attention for CCBT has grown, research on the effectiveness of CCBT for depression in primary care is still in its infancy, especially CCBT offered via the Internet. More research evaluating such interventions is necessary. Therefore, in the current study we will evaluate the (cost-) effectiveness of an online CCBT self-help program for mild to moderate depression in primary care. We will compare CCBT with treatment as usual by a GP, and with a combination of both treatments.

### Why do we need more research on CCBT in primary care?

There are several reasons why we are conducting this study. First, the only study so far on CCBT in primary care used a program that was delivered on a computer in the general practice [26]. Since the Internet can increase the accessibility of such an intervention, we will offer the CCBT program on the Internet. In the Netherlands, almost all of the general adult population has access to Internet, and this will only increase in the next decade [84]. Second, in the only other study on CCBT in primary care [26], a nurse provided practical support at the start and end of each session. We will study the effectiveness of CCBT as a pure self-help intervention; no support or guidance will be given to the patient. Third, the effects of a combined treatment (i.e. both CCBT and treatment as usual by a GP) still need to be evaluated. We think this might have extra effects in terms of improvement in depressive severity and quality of life. Although the GP is not directly involved in the CCBT program, a combined treatment might also increase adherence to the intervention. Previous studies already showed that minimal therapist contact could increase the adherence to Internet-based interventions [85,86], a result which was recently confirmed in a meta-analysis [87]. Next to that, the GP can monitor the progress of the patient and can pay attention to non-verbal signals of the patient. Finally, only one study so far has conducted an economic evaluation of CCBT [27].

### Methodological considerations

Our study has several strengths. First, we will recruit patients from the general population. Unlike in samples selected in general practices or clinics, no biases will occur due to help seeking behaviour of patients and illness recognition by physicians, which is often a problem in depression [10]. Another strength is that we will make full use of the Internet infrastructure by administering all questionnaires online. This reduces the risk of making mistakes while filling out or scoring the questionnaires.

Several limitations of the present study should also be noted. All our outcomes will be measured online and one may question the equality of computerised questionnaires and paper-and-pen versions. However, there are sufficient indications that computerised and paper-and-pen questionnaires show similar construct validity [88,89]. Furthermore, all the outcomes at follow-up will be measured by self-report and as a result information on actual DSM-III diagnoses of depressive episodes at follow-up will be lacking.

## Conclusion

CCBT is a new treatment format with interesting possibilities. It might offer a solution to the current undertreatment of depression. It is a promising treatment for depression in primary care, but more research needs to be done before it can be disseminated and implemented in the current health care system. The current study contributes to the growing literature on the clinical and cost-effectiveness of online CCBT.

## Competing interests

The authors declare that they have no competing interests.

## Authors' contributions

All authors participated in the design of the study. MJHH obtained funding for the study. LEdG and SAHG drafted the manuscript and carry out recruitment and data-collection. All authors contributed to the writing of the manuscript and have approved the final manuscript.

## Pre-publication history

The pre-publication history for this paper can be accessed here:


